# Integration of Deep Learning Network and Robot Arm System for Rim Defect Inspection Application

**DOI:** 10.3390/s22103927

**Published:** 2022-05-22

**Authors:** Wei-Lung Mao, Yu-Ying Chiu, Bing-Hong Lin, Chun-Chi Wang, Yi-Ting Wu, Cheng-Yu You, Ying-Ren Chien

**Affiliations:** 1 Department of Electrical Engineering, Graduate School of Engineering Science and Technology, National Yunlin University of Science and Technology, Yunlin 640301, Taiwan; wlmao@yuntech.edu.tw (W.-L.M.); ice.eye@aandf.com.tw (Y.-Y.C.); katari@aandf.com.tw (B.-H.L.); d11010202@yuntech.edu.tw (C.-C.W.); m10812013@yuntech.edu.tw (Y.-T.W.); m10812014@yuntech.edu.tw (C.-Y.Y.); 2 Department of Electrical Engineering, National Ilan University, Yilan 260007, Taiwan

**Keywords:** robotic arm, rim defect detection, YOLO algorithm, deep convolutional generative adversarial networks (DCGAN)

## Abstract

Automated inspection has proven to be the most effective approach to maintaining quality in industrial-scale manufacturing. This study employed the eye-in-hand architecture in conjunction with deep learning and convolutional neural networks to automate the detection of defects in forged aluminum rims for electric vehicles. RobotStudio software was used to simulate the environment and path trajectory for a camera installed on an ABB robot arm to capture 3D images of the rims. Four types of surface defects were examined: (1) dirt spots, (2) paint stains, (3) scratches, and (4) dents. Generative adversarial network (GAN) and deep convolutional generative adversarial networks (DCGAN) were used to generate additional images to expand the depth of the training dataset. We also developed a graphical user interface and software system to mark patterns associated with defects in the images. The defect detection algorithm based on YOLO algorithms made it possible to obtain results more quickly and with higher mean average precision (mAP) than that of existing methods. Experiment results demonstrated the accuracy and efficiency of the proposed system. Our developed system has been shown to be a helpful rim defective detection system for industrial applications.

## 1. Introduction

Light alloy castings are widely used to reduce the weight of electric vehicles (e.g., wheel rims and steering boxes); however, a high degree of variability in the casting process necessitates careful visual inspection of all such devices. The non-destructive inspection of manufactured items based on computer vision has proven highly effective and efficient; however, the inability of such systems to deal with non-planar objects from multiple angles necessitates manual inspections by human operators, which is expensive and time-consuming.

The automated inspection of tire rims is generally performed using X-ray analysis or conventional image processing [1,2,3]. In the current study, we constructed an automated system to detect defects on the forged aluminum rims of electric vehicles, using deep learning and convolutional neural networks [4,5,6,7]. The proposed system adopted the eye-in-hand architecture, which involves a charge-coupled device (CCD) camera on an ABB robotic arm with a graphical user interface to provide control over the camera trajectory and an adjustable light-emitting diode (LED) lighting system. The captured images are then analyzed using an object detection algorithm. We evaluated the YOLO v3 and YOLO v4deep learning models, both of which are lightweight, unsupervised, and efficient. These networks have previously been used to determine whether a mask has been put on correctly [8], to detect surface defects in the equipment in power substations [9], and to detect ships in aerial radar images [10]. The use of deep learning in a system such as this requires a large number of images presenting flaws of every conceivable type; however, obtaining such images can be difficult. In [11], researchers created adversarial networks (DCGAN, LSGAN, and WGAN) to overcome an insufficient number of images for their training model. GANs and DCGANs have been used to establish systems by which to monitor one-dimensional current waveforms [12]. GANs have been used to increase the accuracy of CNNs for the diagnosis of bladder cancer [13]. DCGANs have been used to expand the dataset of chest X-ray images to enhance classifier performance [14].

In the current study, the images generated using GAN failed to meet our standards; however, DCGAN provided usable results. We therefore combined the original images with photos generated using DCGAN in training YOLO v3 and YOLO v4 and assessed the results. This research contributes to our understanding of detection systems for curved metal surfaces and the application of deep learning networks to detection applications.

The structure of the research work is as described below. Section 2 discusses the overall system architecture. Section 3 describes the related works. Experiments and results are presented in Section 4. Conclusions are presented in Section 5.

## 2. System Design

**Figure 1:**
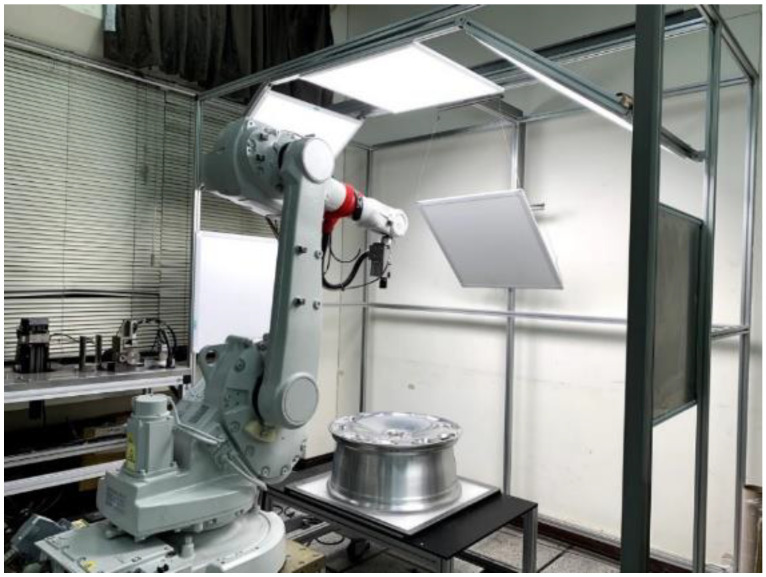
Photographic image showing a practical implementation of the proposed system.

In most existing defect detection systems, the camera(s) is mounted in a fixed position while the workpiece is moved, such that the images used for inspection are aligned vertically relative to the workpiece. Unfortunately, this approach is ill-suited to objects with irregular and/or curved surfaces due primarily to the difficulty of capturing images from multiple angles. In the current study, we adopted the eye-in-hand approach to defect detection, wherein the camera is attached to an ABB robotic arm, and multiple lights are used to provide illumination. A PC-based controller integrates the camera equipment with the control system for the arm. Figure 1 presents an image showing a practical implementation of the proposed system.

**Figure 2:**
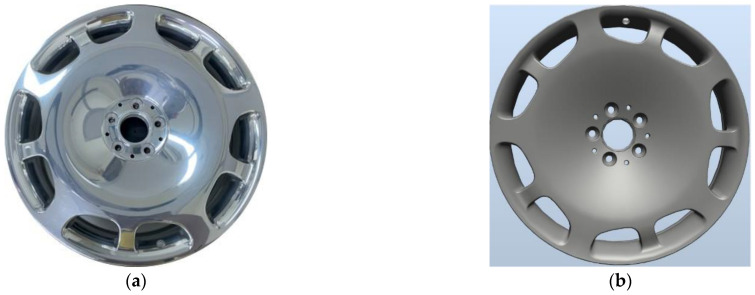
(**a**) Image of actual aluminum rim; (**b**) numerical rendering of the aluminum rim.

**Figure 3:**
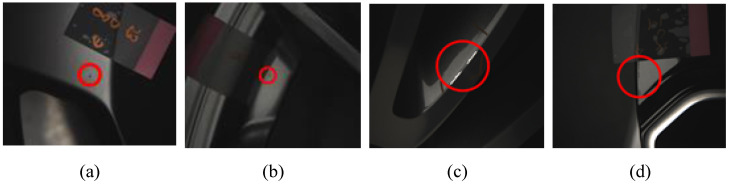
Defects typical of aluminum rims: (**a**) dirt spot, (**b**) paint stain, (**c**) scratch, and (**d**) dent.

The workpiece in the current study was forged aluminum wheel rims (see Figure 2a), a numerical rendering of which is presented in Figure 2b. Forged aluminum wheel rims are subject to a wide range of defects, including dirt spots, paint stains, scratches, and dents, respectively presented in Figure 3a–d.

The imaging system in the current study was based on a color CMOS camera (GS3-U3-51S5C-C; Canada APO Spart) to obtain images at a high sampling rate in real-time (see Table 1).

**Figure 4:**
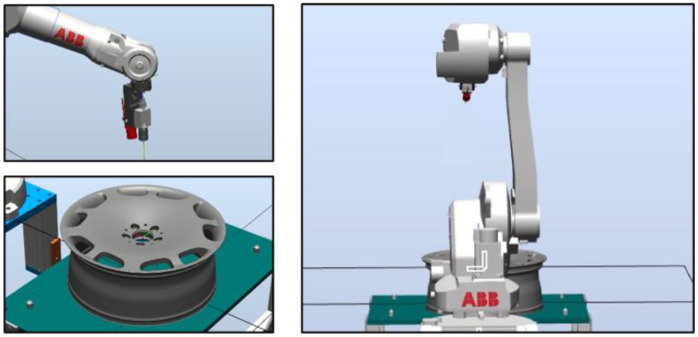
Environment layout.

**Figure 5:**
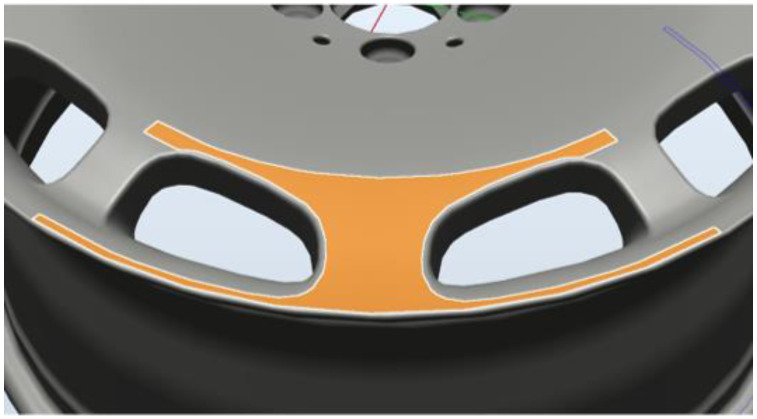
Select the machined surface.

**Figure 6:**
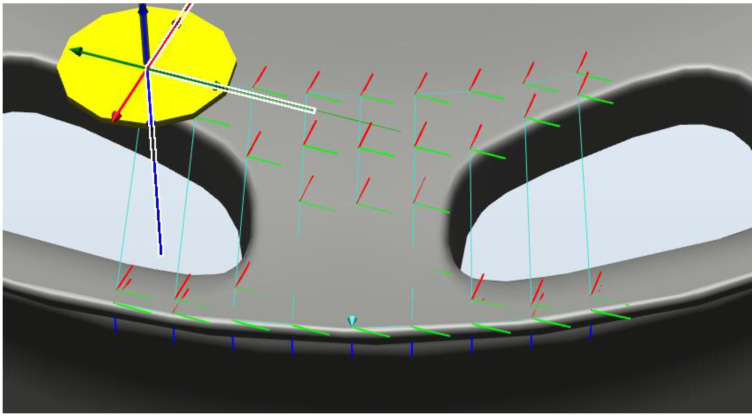
Simulated detection path.

In the following, we outline the methods used to plan the path of the robot arm. In RobotStudio, we first constructed an operating environment, including CAD files of the arm, industrial camera, and wheel rims (see Figure 4). We then created a coordinate map of the tools (camera) and workpiece (wheel rim). We then specified the surface area to be inspected. The resulting generation path was meant to align the camera perpendicular to the surface of interest (see Figure 5). A simulated detection path is presented in Figure 6.

**Figure 7:**
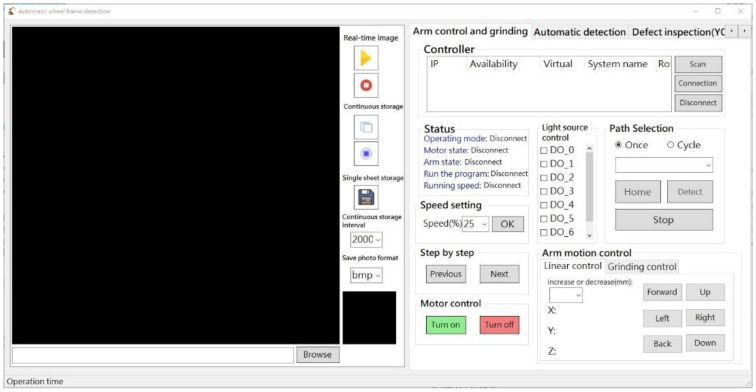
Basic control interface.

**Figure 8:**
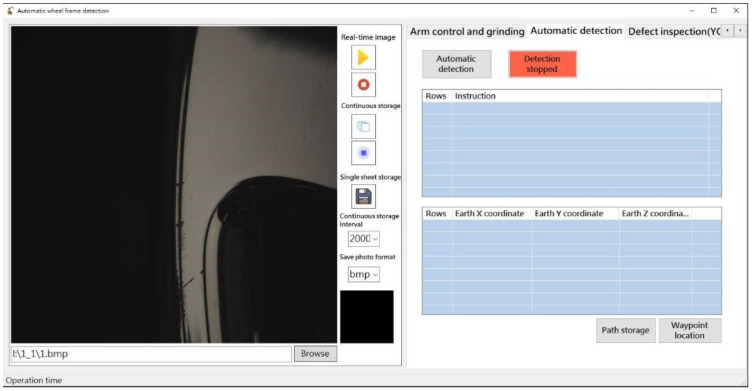
Automated detection interface.

**Figure 9:**
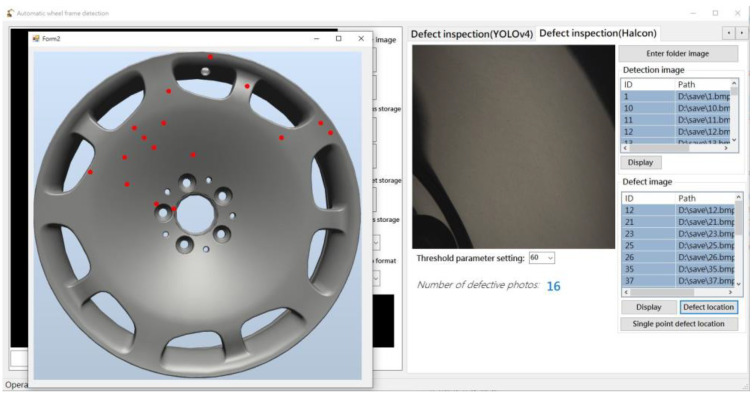
Test results interface.

RobotStudio SDK was used to control the robot arm while displaying real-time operating information and scanning results. In addition, external hardware for image recognition, adjusting imaging parameters, and controlling the multi-angle light source was integrated within the robot arm. Figure 7 presents the basic control interface, Figure 8 presents the automation interface, and Figure 9 presents the test results interface.

**Figure 10:**
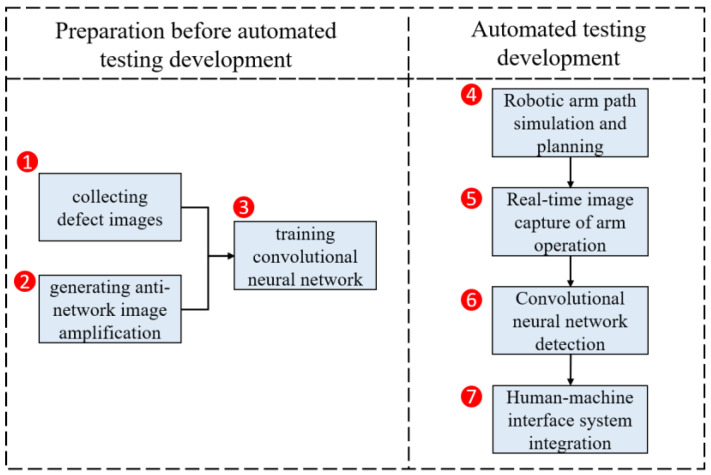
Flowchart showing the experiments conducted in this study.

Our objective in this research was to automate the optical detection of defects in forged wheel rims. Experiments were designed to address (1) the collection of images showing examples of defects, (2) the methods used to expand the training dataset, (3) training of the convolutional neural network, (4) planning and simulation of the robot path, (5) capturing real-time images as the robot arm is moving, (6) algorithmic image analysis, and (7) the human-machine interface. A flowchart of the various experiments is presented in Figure 10.

## 3. Related Works

### 3.1. GAN and DCGAN

**Figure 11:**
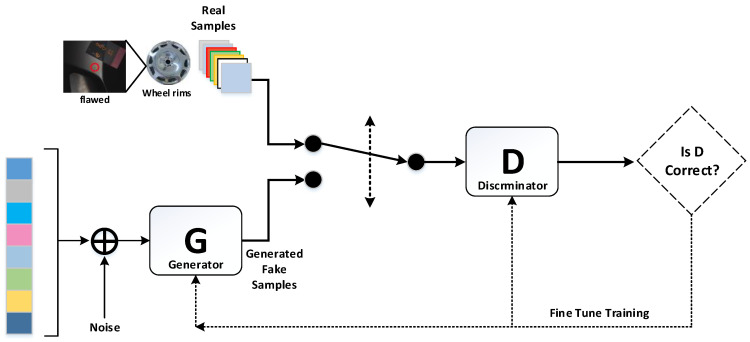
The basic architecture of the GAN network.

Goodfellow et al. [15] developed a framework comprising generative networks and adversarial networks to train two models, including (1) a generator (G) to capture data distributions and (2) a discriminator (D) to differentiate between actual and erroneous defects. The objective of the G model is to maximize the likelihood that the discrimination (D) model will make mistakes. The objective of the D model is to differentiate between actual and erroneous samples. This system iteratively trains both G and D models [16]. Figure 11 presents a schematic diagram showing the basic architecture of an adversarial network.

Discriminator (D) is a binary classifier that classifies data generated by generator (G) as real or unreal. Generator (G) seeks to minimize its loss function based on data classified as real by Discriminator (D). The modeling method is as Equation (1). This means that the objective functions of G and D are inverse (log(D(x)), log(1 − D(G(z))), where z refers to noise with a uniform, normal, or Gaussian distribution. The goal of optimization is to bring the probability distribution of G close to that of D, thereby generating images that resemble actual images of defects. Maximum likelihood estimation (MLE) is used to solve the optimization problem.

(1) $$minmax\left(D,G\right)=E_{x\sim pdata}\left[logD\left(x\right)\right]+E_{z\sim pz}\left[\log\left(1-D\left(G\left(z\right)\right)\right)\right],$$

where x is a real image from the true data distribution pdata; z is a noise vector sampled from distribution pz (e.g., uniform or Gaussian distribution); and training is performed in a minimax game with the global optimum of pz converging to pdata.

Our use of machine learning for the detection of defects requires a large amount of training data corresponding to defects in the real world. However, it is not easy to collect a large number of instances of a given type of defect or to deal with wheels presenting multiple defects. In the current study, we sought to overcome this limitation by generating additional samples using both GAN and DCGAN. In 2016, Radford et al. [17] proposed a DCGAN system in which convolutional neural networks are used for discriminators and generators. Compared to the original GAN, DCGAN provides superior stability, ease of convergence, and image samples of superior quality. The architecture of DCGAN is comparable to that of GAN; however, both the generator and the discriminator use convolutional neural networks. In each convolutional layer, batch regularization is applied to the generator and discriminator to enhance stability.

### 3.2. YOLO v3 and v4

When dealing with deep neural networks, training effectiveness depends on depth. Prior to the development of ResNet [18], increasing the number of training layers often led to gradient disappearance or explosion, which could seriously compromise accuracy. In 2018, Redmon and Farhadi [19] updated YOLO (version v3), using ResNet to resolve the problem of gradient disappearance and explosion in conjunction with multi-scale feature maps to enhance detection and predictive performance for small objects [20].

**Figure 12:**
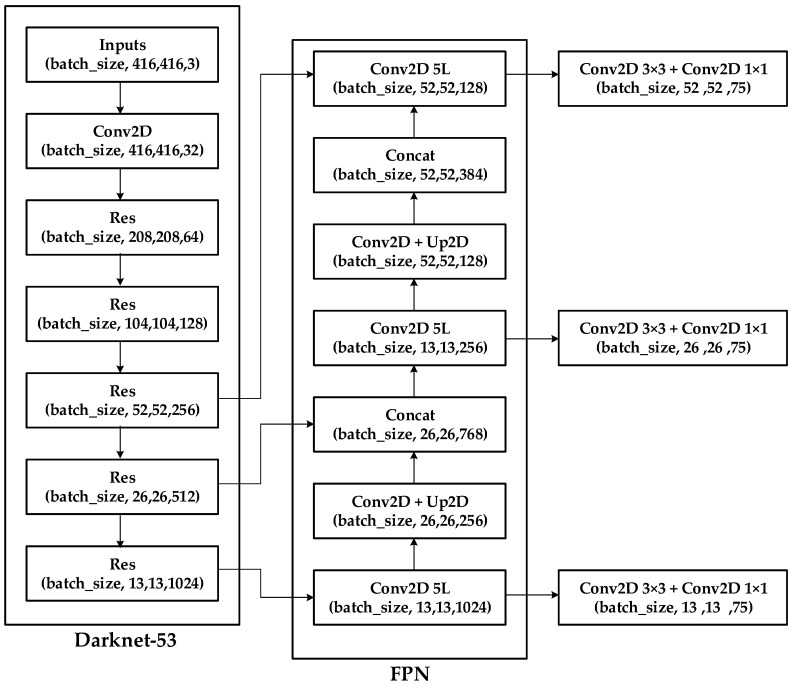
Schematic diagram showing the architecture of YOLO v3.

YOLO v3 employs the feature pyramid network (FPN) architecture, which uses multi-scale feature mapping to facilitate the detection of objects. For example, a 416 × 416 image might undergo downsampling 32 times, 16 times, and eight times to obtain feature maps at three different scales. Figure 12 illustrates the architecture of YOLO v3 [21].

**Figure 13:**
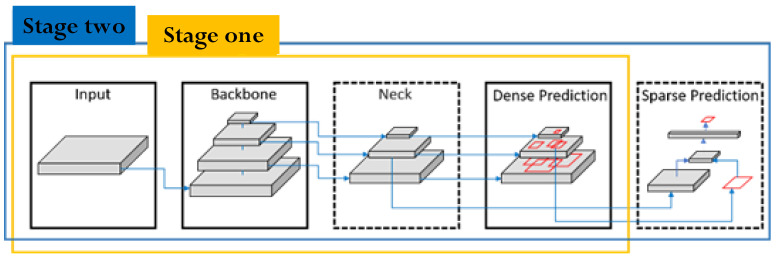
Schematic diagram showing the YOLO v4 object detection architecture.

In 2020, Bochkovskiy et al. [22] developed YOLO v4 based on numerous detection optimization schemes analysis. The resulting algorithm uses fewer parameters in the main network to enhance calculation speed and recognition accuracy. Figure 13 shows the architecture of YOLO v4.

#### 3.2.1. Input

YOLO v4 uses the Mosaic method for image amplification, which involves the zooming, cropping, and stitching of four photos extracted from the input dataset.

#### 3.2.2. Backbone

YOLO v4 represents an attempt to improve the operating speed of neural networks by implementing the Cross Stage Partial Network (CSPNet) [23] structure using fewer convolution groups in the convolutional layer (1–8 groups) and then combining CSPNet with ResNeXt50 and Darknet53. This network architecture was shown to enhance the learning ability of CNNs with a corresponding effect on prediction accuracy while eliminating computational bottlenecks to reduce memory usage.

#### 3.2.3. Neck

YOLO v4 employs Spatial Pyramid Pooling technology [24] and Path Aggregation Network technology [25] for optimization in the Neck, intending to fuse local and global features to improve the results obtained using the final feature map. Essentially, this involves combining four feature maps of different scales to expand the horizon of perception.

#### 3.2.4. Head

In the Head, YOLO v4 adopts the predictive framework of YOLO v3, wherein the creation of a bounding box is based on offset and confidence levels. The backbone is based on the smooth, continuous, self-regularized, and non-monotonic Mish activation function [26]:

(2) $$f\;\left(x\right)=xtanh\left(softplus\left(x\right)\right)=xtanh\left(\ln\left(1+e^{x}\right)\right)$$

The features of YOLO v4 are outlined in the following:

(1) Bag of Freebies (BoF) for backbone: CutMix [27] and Mosaic are used for data augmentation, whereas DropBlock [28] and Class label smoothing [29] are used to avoid overfitting regularizers.
(2) BoF for detector: Complete intersection over union loss (CIOU loss) is used to improve convergence accuracy, while cross mini-batch normalization (CmBN) is used to reduce the computational burden, and self-adversarial training (SAT) is used for data enhancement [9], and DropBlock and Mosaic are used for data augmentation.
(3) Bag of Specials (BoS) for backbone: CSPNet is used to improve accuracy and reduce memory usage and implement the Mish activation function and multi-input weighted residual connections (MiWRC).
(4) BoS for detector: A spatial attention module (SAM-block) is used to improve training efficiency in implementing distance intersection over union (DIoU-NMS), the SPP-block, the PAN path-aggregation block, and the Mish activation function.

## 4. Experimental Results

### 4.1. Collecting a Dataset of Images Showing Manufacturing Flaws

**Figure 14:**
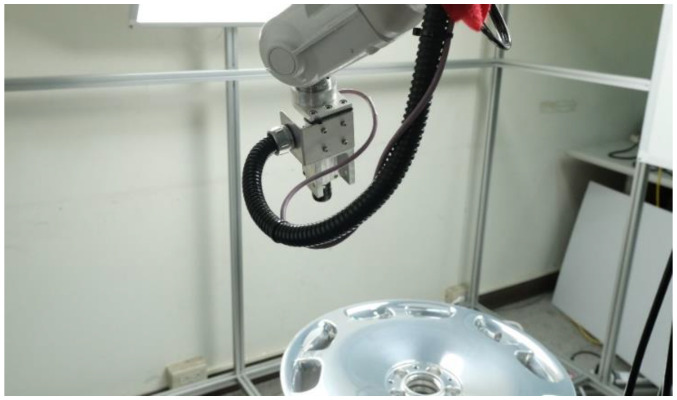
Practical implementation of the proposed defect detection system.

Our objective was to improve detection accuracy by making the defects large, diverse, and distinct from the background to facilitate the training of the convolutional neural network. Figure 14 presents a photographic image showing the practical implementation of the proposed defect detection system.

**Figure 15:**
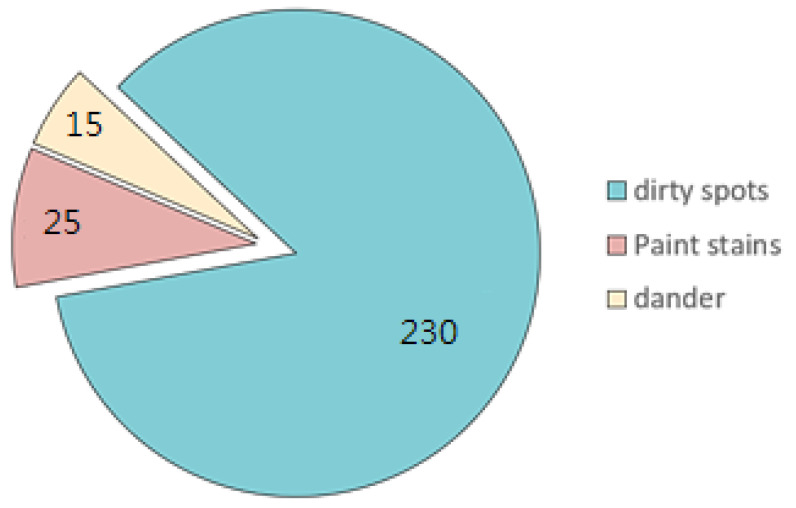
Distribution of defect types as percentages.

**Figure 16:**
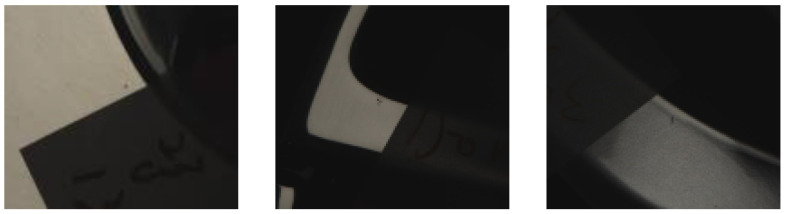
Photographs examples of the three types of defect addressed in this study.

Most of the rims used in this study had dirt spots and/or paint stains. From these actual rims, we collected 245 images of defects. We compiled a total of 270 defects, including 230 dirt spots, 25 paint stains, and 15 dander defects. Figure 15 illustrates the distribution of defect types as percentages. Figure 16 presents examples of the three types of defects.

### 4.2. Image Dataset

Automated systems designed to detect defects require a large number of samples to achieve high recognition performance. Unfortunately, in the real world, assembling a dataset of sufficient size can be exceedingly difficult. We used GAN and DCGAN to generate images showing simulated defects in the current study. Generative models are meant to generate a diverse set of images that closely resemble actual samples to augment the training dataset.

### 4.3. Image Augmentation and Scaling

**Figure 17:**
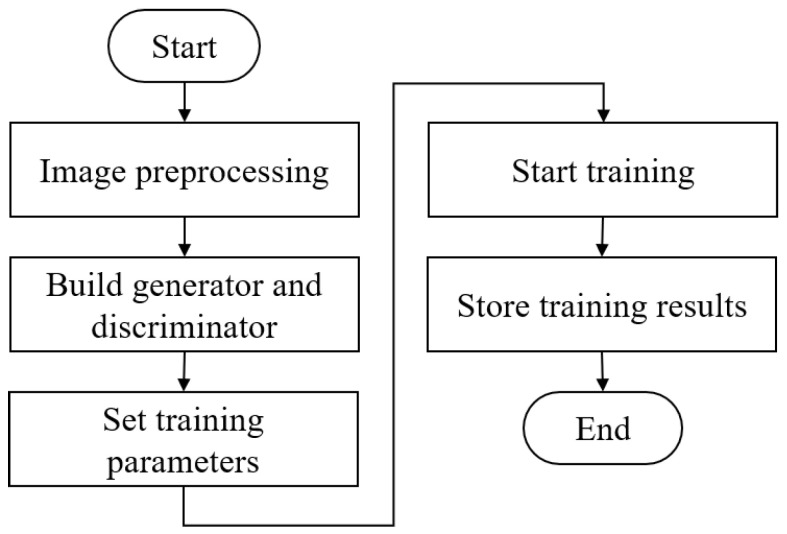
Flowchart showing the implementation of the proposed generative adversarial networks.

Images measuring 2448 × 2048 were reduced to dimensions suitable for the generative network (i.e., 270 images measuring 64 × 64). We employed the open-source library Keras to create generative adversarial network models (GAN and DCGAN) for use in generating images by which to train YOLO. Figure 17 presents a flowchart of the generative adversarial network.

**Figure 18:**
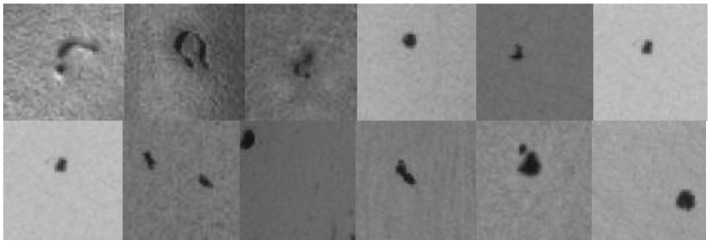
Examples of cropped flawed images.

When the 2448 × 2048 image is directly input to the neural network, the output will not highlight the characteristics of the flaw. First, we use the image processing software to crop the flawed images to a size of 64 × 64, as shown in Figure 18 below. Then, the 270 pieces of 64 × 64 flaw images are stored in the dataset.

After importing the required packages, libraries, and image input dimensions into GAN and DCGAN, we set the number of iterations and batch size for model training. Note that there is no set standard for the number of iterations or batch size; however, the batch size must not exceed the memory capacity. Note also that the size of these parameters is proportional to the time required for training.

### 4.4. Training Results

**Figure 19:**
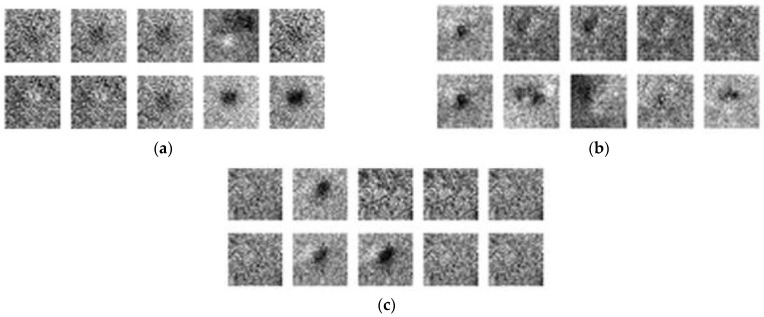
GAN training results: (**a**) 10,000 iterations, (**b**) 20,000 iterations, and (**c**) 30,000 iterations.

As shown in Figure 19a, after running the GAN model through 10,000 iterations, the flaws in the images began to take shape. Running 20,000 iterations (Figure 19b) or 30,000 iterations (Figure 19c) did not significantly affect the output images, which indicates that the GAN model was unable to reach convergence when applied to this training dataset.

**Figure 20:**
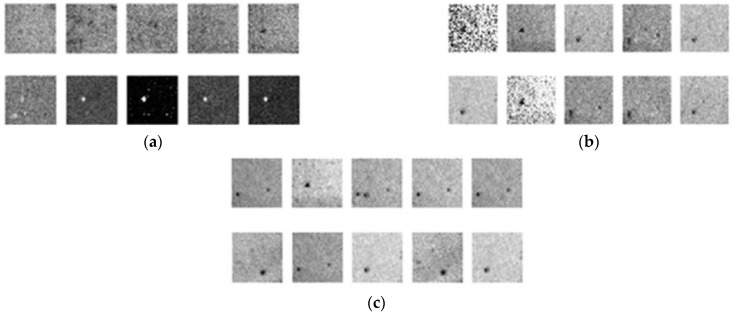
DCGAN training results: (**a**) 10,000 iterations; (**b**) 20,000 iterations; (**c**) 30,000 iterations.

Figure 20a, 20b, and 20c respectively present the results of DCGAN after 10,000, 20,000, and 30,000 iterations. After 10,000 iterations, the image has gradually become a prototype. After 20,000 iterations, the noise began to interfere with the features of the defects; however, the outline of the defects remained discernable. After 30,000 iterations, the flaws are easily discerned, and the images with less noise are indistinguishable from the original samples, which indicates that DCGAN achieved convergence.

**Figure 21:**
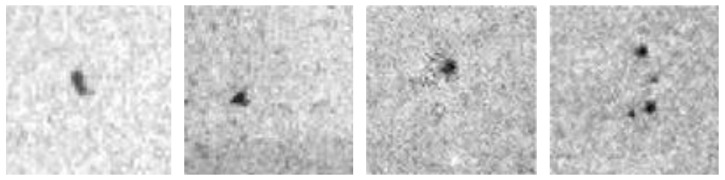
Comparison of actual photographic images and generated images.

A comparison of the images generated using DCGAN (30,000 iterations) and actual images (Figure 21) revealed that the proposed dataset augmentation scheme was highly effective in generating a diversity of realistic defects. Generated images (640 × 480) were then stored for use in training YOLO.

### 4.5. Training the Convolutional Neural Network

**Figure 22:**
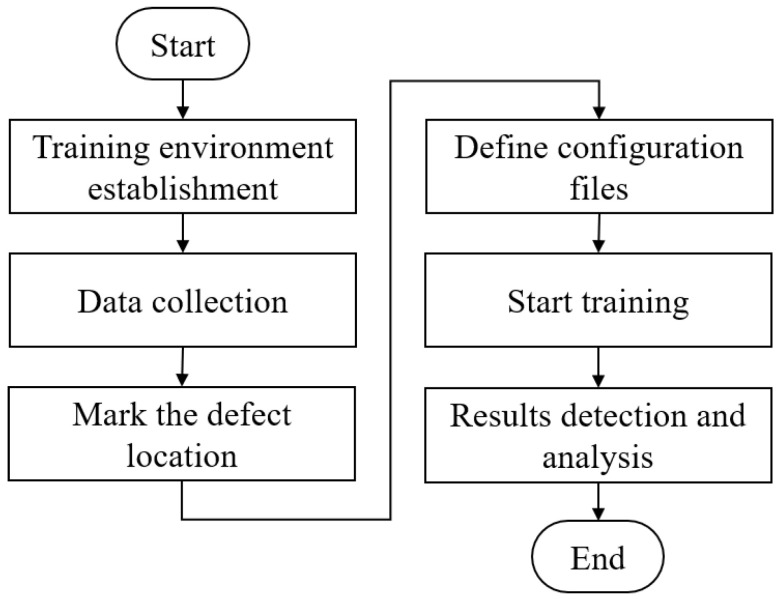
Flowchart showing experiments involving the application of original and generated images to YOLO v3 and YOLO v4.

We respectively trained YOLO v3 and YOLO v4 using the original and DCGAN-generated images. We then evaluated the four sets of training results in order to identify the best image dataset (original images or generated images). We also sought to identify the best network architecture for defect detection (YOLO v3 or YOLO v4). The training process is illustrated in Figure 22.

We organized the training samples and annotation files to create a dataset for training. The distribution of flaws was as follows: dirt spots (85%), paint stains (9%), and dander defects (6%). Note, however, that despite the nature of the defects, they appeared quite similar to dirt smears. This allowed us to merge the three types of the defect into a single classification category, hereafter referred to as a defect. Table 2 lists the details of datasets used in the four evaluations.

### 4.6. CNN Detection Results

**Figure 23:**
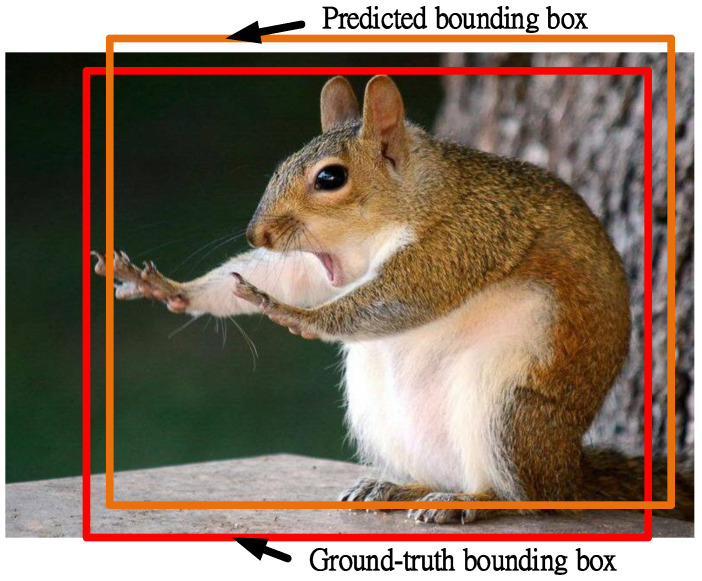
Schematic diagram illustrating the predicted and actual bounding boxes.

The detection results were evaluated using the mean average precision (mAP) in model recognition and a confusion matrix. The concept of mAP is similar to that of Intersection over Union (IoU). Based on the schematic diagram in Figure 23, the *IoU* of sets A and B can be calculated as follows:

(3) $$IoU\left(A,B\right)=\frac{A\cap B}{A\cup B},$$

where set A denotes the predicted bounding box and set B indicates the ground-truth bounding box.

The confusion matrix comprised the following four elements:

- True Positive (TP): Correctly identified positive samples.
- True Negative (TN): Correctly identified negative samples.
- False Positive (FP): Incorrectly identified as positive samples (type-I error).
- False Negative (FN): Incorrectly identified as negative samples (type-II error).

After defining the four elements, we assessed the quality of the model by deriving the corresponding *Accuracy*, *Recall*, and *Precision* as follows:

(4) $$Accuracy=\frac{TP+TN}{\left(TN+TP+FN+FP\right)}$$

(5) $$Recall=\frac{TP}{\left(TP+FN\right)}$$

(6) $$Precision=\frac{TP}{\left(TP+FP\right)}$$

**Figure 24:**
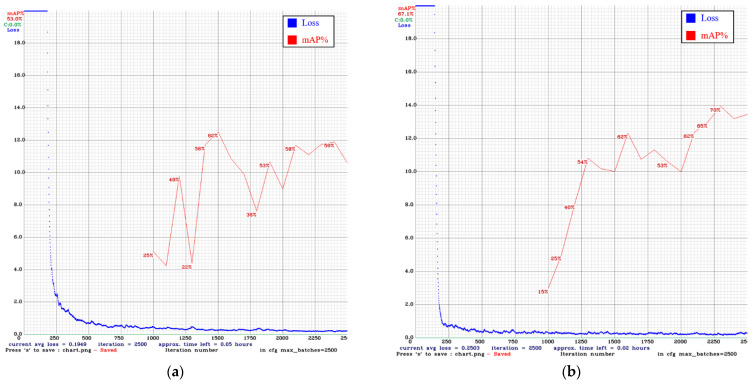
Training results for YOLO v3: (**a**) original images only (**b**) original images plus DCGAN synthetic images.

**Figure 25:**
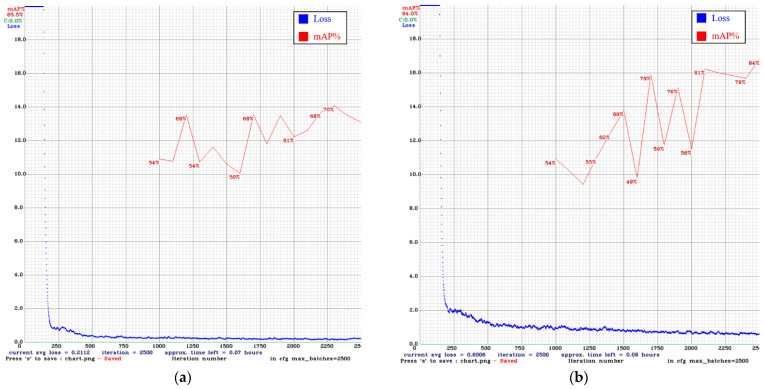
Training results for YOLO v4: (**a**) original images only (**b**) original images plus DCGAN synthetic images.

In Figure 24 and Figure 25, the blue line is the loss curve, and the red line is the mAP. In our comparison of models after 2500 iterations, the mAP of YOLO v3 using only the original images was 53.0%. Adding DCGAN images increased the mAP to 67.1%. The mAP of YOLO v4 using only the original images was 65.5%. Adding DCGAN images increased the mAP to 84.0%. Overall, YOLO v4 outperformed YOLO v3, and the inclusion of synthetic images further improved performance.

**Figure 26:**
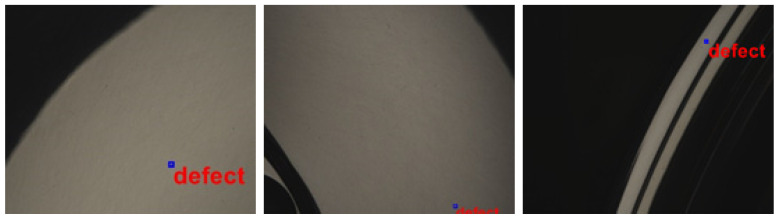
Photographs showing the locations of defects.

The second stage of testing was performed using eight rims, comprising 25 defects. Model prediction data are listed in Table 3, and the calculation results are listed in Table 4. In the test results in Figure 26, defects are indicated by boxes.

We conducted further analysis of the best training model (YOLO v4+DCGAN) to determine whether increasing the number of iterations would increase detection accuracy. The results are listed in Table 5. The times required for the system to complete the inspections are listed in Table 6.

We compared two image recognition algorithms and two image generation algorithms in this experiment. Overall, we determined that DCGAN was superior to the conventional GAN. The proposed system using YOLO v4 plus DCGAN achieved an accuracy of 86.8%. Furthermore, running the system through 20,000 iterations provided optimal results in terms of detection accuracy with no significant increase in computation time.

## 5. Conclusions

This paper presents an automated system for the detection of defects on irregular curved surfaces of aluminum, which are generally poorly suited to optical analysis. We overcame these limitations using a multi-angle image capture scheme with multiple adjustable light sources. We also assessed the performance of the system using the YOLO v3 and YOLO v4 deep learning models.

Wheel rims can have as many as 90 types of flaws; however, it is difficult to obtain a sufficient volume of training data for every type of flaw. We, therefore, used GAN and DCGAN to enable the generation of additional images to augment the sparse datasets. This approach proved highly effective in enhancing the accuracy, recall, and precision ratios of YOLO v3 (+6.5%) and YOLO v4 (+37.7%).

## Figures and Tables

**Table 1:** Specifications of the industrial camera GS3-U3-51S5C-C.

Firmware	2.25.3	Gain Range	0 dB~48 dB
Resolution	2448 × 2048	Exposure Range	0.006 ms~32 s
Frame Rate	75 FPS	Interface	USB3.1
Chrome	Color	Dimensions/Mass	44 mm × 29 mm × 58 mm/90 g
Sensor	Sony IMX250, CMOS,2/3”	Power Requirements	5 V via USB3.1 or 8~24 V via GPIO
Readout Method	Global shutter	Lens Mount	C-mount

**Table 2:** Dataset details used to evaluate image sets and CNNs.

Experiment\Total Sample	Total Number of Samples (Photos)	Number of Training Samples (Photos)	Number of Testing Samples (Photos)
YOLO v3 Original images	245	196	49
YOLO v4 Original images	245	196	49
YOLO v3 Original images + DCGAN	545	436	109
YOLO v4 Original images + DCGAN	545	436	109

**Table 3:** Model prediction data.

Analysis\Methods	YOLO v3	YOLO v4	YOLO v3 + DCGAN	YOLO v4 + DCGAN
TP	217	98	176	213
FP	268	67	153	56
FN	89	209	130	93
TN	562	770	677	774

**Table 4:** Calculated results.

Analysis\Methods	YOLO v3	YOLO v4	YOLO v3 + DCGAN	YOLO v4 + DCGAN
Total number of defects	306	307	306	306
detected	217	98	176	213
Accuracy	68.5%	75.8%	75%	86.8%
Recall	70.9%	31.9%	57.5%	69.6%
Precision	44.7%	59.3%	53.4%	79.1%

**Table 5:** Detection accuracy as a function of the number of iterations.

Methods\Analysis	Accuracy	Precision	Recall
YOLO v4 + DCGAN (5000)	80.6%	66.4%	56.2%
YOLO v4 + DCGAN (4000)	63.7%	41.1%	80.0%
YOLO v4 + DCGAN (3000)	76.1%	54.4%	70.2%
YOLO v4 + DCGAN (2000)	86.8%	79.1%	69.6%

**Table 6:** Computational efficiency of the proposed automated detection system.

Methods\Time	Robot	detect	Total
YOLO v3	2 min 39 s	56.3 s	3 min 35.3 s
YOLO v3 + DCGAN	2 min 39 s	56.2 s	3 min 35.2 s
YOLO v4	2 min 39 s	56.3 s	3 min 35.3 s
YOLO v4 + DCGAN(5000)	2 min 39 s	56.1 s	3 min 35.1 s

## Data Availability

Not applicable.
